# Modeling the Cost Effectiveness of Malaria Control Interventions in the Highlands of Western Kenya

**DOI:** 10.1371/journal.pone.0107700

**Published:** 2014-10-07

**Authors:** Erin M. Stuckey, Jennifer Stevenson, Katya Galactionova, Amrish Y. Baidjoe, Teun Bousema, Wycliffe Odongo, Simon Kariuki, Chris Drakeley, Thomas A. Smith, Jonathan Cox, Nakul Chitnis

**Affiliations:** 1 Department of Epidemiology and Public Health, Swiss Tropical and Public Health Institute, Basel, Switzerland; 2 University of Basel, Basel, Switzerland; 3 Faculty of Infectious and Tropical Diseases, London School of Hygiene and Tropical Medicine, London, United Kingdom; 4 Centre for Global Health Research, Kenya Medical Research Institute/Centers for Disease Control and Prevention, Kisumu, Kenya; 5 Johns Hopkins Malaria Research Institute, Johns Hopkins Bloomberg School of Public Health, Baltimore, Maryland, United States of America; 6 Radboud University Nijmegen Medical Centre, Nijmegen, the Netherlands; Université Pierre et Marie Curie, France

## Abstract

**Introduction:**

Tools that allow for in silico optimization of available malaria control strategies can assist the decision-making process for prioritizing interventions. The OpenMalaria stochastic simulation modeling platform can be applied to simulate the impact of interventions singly and in combination as implemented in Rachuonyo South District, western Kenya, to support this goal.

**Methods:**

Combinations of malaria interventions were simulated using a previously-published, validated model of malaria epidemiology and control in the study area. An economic model of the costs of case management and malaria control interventions in Kenya was applied to simulation results and cost-effectiveness of each intervention combination compared to the corresponding simulated outputs of a scenario without interventions. Uncertainty was evaluated by varying health system and intervention delivery parameters.

**Results:**

The intervention strategy with the greatest simulated health impact employed long lasting insecticide treated net (LLIN) use by 80% of the population, 90% of households covered by indoor residual spraying (IRS) with deployment starting in April, and intermittent screen and treat (IST) of school children using Artemether lumefantrine (AL) with 80% coverage twice per term. However, the current malaria control strategy in the study area including LLIN use of 56% and IRS coverage of 70% was the most cost effective at reducing disability-adjusted life years (DALYs) over a five year period.

**Conclusions:**

All the simulated intervention combinations can be considered cost effective in the context of available resources for health in Kenya. Increasing coverage of vector control interventions has a larger simulated impact compared to adding IST to the current implementation strategy, suggesting that transmission in the study area is not at a level to warrant replacing vector control to a school-based screen and treat program. These results have the potential to assist malaria control program managers in the study area in adding new or changing implementation of current interventions.

## Introduction

Important progress has been made in the past decade in reducing malaria morbidity and mortality in Kenya, but it is not obvious which additional tools and strategies should be the next priority to include in the package of malaria control interventions in a given area to keep transmission levels low, especially given the threat of resistance of the parasite and vectors to antimalarial drugs and insecticides [1], [2]. Application of mathematical models for use in simulations of malaria epidemiology and control can help estimate the impact of interventions singly and in combination to support this goal.

OpenMalaria, a stochastic simulation modeling platform [3], has previously been applied to Rachuonyo South District, Nyanza Province, Kenya in order to describe the epidemiology of malaria and control area and identify uncertainty in key parameters pertaining to the study area [4]. Results indicate that the OpenMalaria model, as parameterized for Rachuonyo South District, can be extended to simulate the epidemiologic and economic impact of combinations of a range of existing and potential future malaria control interventions, singly and in combination, implemented in the study area [4]. This study addresses the cost effectiveness of feasible malaria control interventions in Rachuonyo South District for a five year time horizon.

### Study area

Rachuonyo South District in Homa Bay County of Nyanza Province, Kenya is a highland fringe area with altitude between 1,400 and 1,600 meters. Ethnicity is predominantly Luo and homesteads are distributed broadly across a rolling landscape intersected with small streams and rivers. The area is characterized by generally low malaria endemicity with marked seasonal and inter-annual variations in transmission [5], [6]. As a result of a 2009 survey, community level parasite prevalence was estimated to be 4.5% and transmission was measured with an entomological inoculation rate of 1.5 infectious bites per person per year [4], but subsequent surveys in the study area showed community-level parasite prevalence to be as high as 15.5% [7]. This is in the range of the reported 2010 national average parasite prevalence of 12%, but low compared prevalence in the neighboring lowland districts bordering Lake Kisumu that reach 38% in children under 15 [8]. Malaria transmission peaks twice each year following rainfall patterns with a long rainy season between March and June and a shorter season in October and November. Recent studies indicate that *Plasmodium falciparum* is transmitted not only by *Anopheles funestus* and *An. arabiensis*, but also by another, as yet unidentified secondary vector with outdoor-active, early-biting behavior, potentially challenging the effectiveness of current vector control interventions targeting indoor-biting mosquitoes [9].

The main malaria control methods are currently mass-distribution of LLINs, annual indoor residual spraying (IRS) with pyrethroids, and prompt and effective treatment [8], [10], [11]. Kenya's health system relies heavily on user fees and other out-of-pocket payments, with exemptions for children under five, the poor, and special conditions and services such as malaria and tuberculosis, in both the formal public and private sector [12]. The latter provides a substantial proportion of primary care services (31%) [13].

Rachuonyo South is one of a number of field sites of the Malaria Transmission Consortium (MTC), a project with the goal of enabling operational program managers to achieve optimal implementation of transmission-reducing malaria control techniques. Active between 2009 and 2012, MTC surveys provided detailed entomological studies of species composition and biting behavior [9], transmission estimation and community evaluation of LLINs and IRS versus LLINs alone. To complement these studies, a trial to assess the effect of hotspot-targeted interventions in populations living both inside and outside hotspots has recently been implemented [14]. Targeted interventions of this trial included distribution of LLINs, IRS, larviciding and focal screening and treatment.

## Methods

### Ethics approval

The study proposal received ethics approval from the Ethical Review Committee (ERC) of the Kenya Medical Research Institute (KEMRI) Nairobi under proposal number SSC 2163, the London School of Hygiene & Tropical Medicine ethics committee (#6111), and from Centers for Disease Control and Prevention (with exempt status).

### OpenMalaria modelling platform

A team at the Swiss Tropical and Public Health Institute (Swiss TPH) and Liverpool School of Tropical Medicine (LSTM) developed the OpenMalaria platform comprising stochastic simulation models of transmission of malaria based on the simulation of infection in individuals. These models are able to evaluate the impact (cost-effectiveness, clinical, epidemiological and entomological) of numerous intervention strategies for malaria control [3], [15]–[19]. The details of the methods to build and parameterize the transmission model used in this project have been published elsewhere [3], [15]–[19]. Briefly, individual infections in humans are simulated by stochastic series of parasite densities, which determine an individual's morbidity and mortality risks as well as their infectiousness to vectors [3], [15]. These simulated infections are linked to a model of transmission of malaria between humans and mosquitoes and to models of interventions [3], [15], [16].

### Model parameterization and experiment design

The scenario describing the current intervention mix was parameterized using a previously-published model of malaria epidemiology and control in Rachuonyo South District, validated with observed data from the site-specific MTC studies described above [4]. Parameterization of this baseline scenario included the characteristics of vector composition and biting behavior, seasonality of transmission, treatment seeking behavior and existing malaria control interventions in the study area as described above.

Combinations of interventions for the experiment were chosen in collaboration with malaria control personnel in the study area to correspond to a 2011–2012 intervention evaluation trial [14]. LLIN use the previous night was simulated at the proportion observed in the population (56%) and an increased level (80%) with one mass distribution at the beginning of the study period. Proportion of houses receiving IRS with a pyrethroid was simulated at the proportion observed in the population (70%) and an increased level (90%). The implementation schedule for IRS was simulated at the observed once-yearly schedule of alternating start dates in April and then June, as well as consistent implementation starting in April, May, and June. Intermittent screen and treatment of school aged children with Artemether lumefantrine (AL) was simulated at low (40%) and high (80%) coverage, and a frequency of either once (January, May and September) or twice (initial months plus March, July and November) per school term. These combinations, as well as their coverage levels and implementation schedules, are described in **Table 1**.

**Table 1:** Experiment design of the combinations and coverage levels of interventions simulated for the study.

	LLIN use (%)	IRS coverage (%)	IRS deployment month	School-based IST coverage (%)	IST frequency (per school term)	Fevers receiving an antimalarial (%)
**Current strategy** *	56	70	Alternating April/June			28
**No intervention**						28
**Increase coverage**	80	90	Alternating April/June			28
**Add school-based IST**	56	70	April	80	2	28
	80	90	April	80	2	28
**Change timing of IRS**	56	70	April			28
	56	70	May			28
	56	70	June			28
**Change timing and increase coverage of IRS**	56	90	April			28
	56	90	May			28
	56	90	June			28
**IRS alone, change coverage**		70	Alternating April/June			28
		90	Alternating April/June			28
**LLINs alone**	56					28
	80					28
**IST alone**				40	1	28
				40	2	28
				80	1	28
				80	2	28

*Represents the base case scenario as parameterized in Stuckey et al. 2012 [4].

### Model Implementation

Each intervention strategy was simulated in a population of 100,000 individuals. To simulate the status quo prior to interventions, simulations were run for one human life span to induce an “equilibrium” level of immunity. Forward simulations of each intervention combination were made using an ensemble of 14 model variants for malaria in humans to address model uncertainty [18], with each model variant repeated with five random seeds to address stochasticity. Each intervention combination was simulated for a period of five years assuming 28% of fevers receive an antimalarial [8]. Simulations were run over the malariacontrol.net volunteer computing platform (www.malariacontrol.net).

### Estimating the cost of malaria case management and interventions

#### Case management costing model

Malaria case management costs were based on a societal perspective; direct costs to the health systems are considered, as well as direct expenditures associated with malaria episodes at the household level. Indirect costs, including productivity loss due to illness, were not accounted for. While the latter tend to dominate the economic cost of illness [20]–[23], including these in cost-effectiveness analysis would result in double-counting of intervention benefits [24]–[26].

Treatment costs are evaluated following a model of malaria case management developed for endemic settings and is described elsewhere [25]. Briefly, the entry point to the model is an acute malaria episode from where treatment seeking is described in terms of the formal and informal sector, and then by level of care compliance with the recommended first-line antimalarial, and further by type of treatment and adherence and drug quality of that treatment. Defined in this manner, the methodology captures patterns in health seeking behavior in a given setting that reflect the underlying health systems infrastructure, quality of health care delivery as well as individual preferences and beliefs about and understanding of clinical outcomes associated with the illness. The methodology to evaluate effectiveness of malaria service delivery using data from national surveys and literature is detailed elsewhere [27]. While the proportion of fevers in Kenya that access medical care is estimated at 61.8% based on demographic health and surveillance (DHS) data [28], effective coverage will be much lower due to poor adherence to drug regimen, intake of counterfeit antimalarials, and drug resistance [27].

On the provider side, cost per episode covers drugs, diagnosis, medical personnel, facility charges, and other consumables. In addition to the first-line antimalarial as per national malaria guidelines, a portion of uncomplicated cases were assigned to treatment with sulfadoxine pyrimethamine (SP) given evidence on moderate uptake of AL, the first line artimisinin combination therapy (ACT) in the study area [29], [30]. Drug costs associated with severe illness include intravenous and oral quinine, with length of regimen varied by outcome. Kenya's national policy of treating severe illness with intra venous (IV) artesunate had not been implemented at the time of the study and was therefore not included in the costing model. For hospitalizations leading to recovery, costs included an initial dose of IV quinine, followed by three further days of IV quinine and four days oral quinine. For severe cases that develop into neurological sequelae, costs included an initial dose of IV quinine followed by 4.5 days of further IV quinine treatment, and subsequent 5.5 days of oral quinine therapy. Severe fatal events were assumed to occur within 48 hours of hospital admission and therefore involve only the initial loading dose of IV quinine and two more days of IV quinine treatment [31]. Drug costs were calculated according to age and weight appropriate regimens [22]. Costs of diagnosis with RDT were calculated proportionally to the fraction of fever cases tested. Facility, personnel, linens, consumables and other outpatient “hotel” charges were obtained from the WHO-CHOICE project [32]. Costs by facility type including health centers with beds, health centers with no beds, and hospital outpatient and inpatient departments were then matched with respective probability of seeking care at a given level estimated from the 2009 Kenyan DHS survey [28]. The DHS patterns in health seeking behavior for febrile illness are likely representative of uncomplicated malaria in countries with high levels of transmission, and somewhat biased in countries with low EIR to the extent that mothers are able to differentiate malaria from other febrile illnesses and care for their children differently. For severe episodes treated in inpatient settings, facility charges were scaled to account for length of hospitalization: 4.5 days for severe episodes that recover, 10 days for severe episodes that develop into neurological sequelae, and 2 days for terminal episodes [31]. Costs were inflated to 2012 using the average annual CPI estimated over the 2008–2011 year period [33] and can be found in **Text S1**.

Direct patient costs associated with a malaria episode include travel expenses to and from healthcare facility and other consumables (i.e. water, food, etc) and were based on the multi-country literature review. Spending on consumables is generally considered negligible; only a few studies recorded these data with an average of $0.20 per visit [25], [34], [35]. For treatment outside of the formal sector including pharmacy, shop, and other sources of care based on self-diagnosis, it is assumed that patients do not incur any additional costs to purchase the drug because these providers are generally close to the patient's home. Thus only drug costs were added for treatments in informal sector.

Both average and marginal health system costs were calculated for each outcome. The average cost includes all costs involved in delivering a health intervention, including the use of spare capacity or slack in the system, health care resources diverted from other uses, and existing health sector resources shared with other health programs. In the marginal analysis only costs of drugs, diagnosis, and patient spending per visit were considered, as broader savings to the health system including labour and capital costs would not be immediately affected by changes in consumption of medical services due to lower diseases burden achieved by control interventions [31], [36].

A sensitivity analysis was conducted for the costs of test and cost per ACT dose by varying costs −50%/+100%, and for proportion of fevers that access medical care by varying access −/+50% (**Table 2**).

**Table 2:** Costing and sensitivity analysis of the Kenya public sector case management system.

			Sensitivity analysis	
Parameter	Unit	Value per unit	Lower value	Upper value
**Cost per test**	Paracheck® rapid diagnostic test	$0.62 [38]	$0.31	$1.24
**Cost per tablet, uncomplicated treatment**	Coartem® (Artemether-lumefantrine)	$0.0898 [54]	$0.045	$0.1769
**Access to treatment**	Proportion of the most recent episode of fevers in children under five within 2 week recall seeking medical care	0.6183 [28]	0.309	0.927

All costs are in 2012 USD.

#### Costing interventions

A general approach for costing malaria interventions using secondary data was applied as outlined by Kolaczinski et al [37]. Current cost of commodities including LLINs, insecticide, and drugs were sourced from the Global Fund to Fight AIDS, Tuberculosis and Malaria Price and Quality Reporting Tool [38]. Costs associated with delivery of interventions and intervention mixes were estimated by reviewing Kenyan field trials predominately from around the study area as identified in a recent systematic review of costs of malaria interventions [39] (**Table 3**). These non-tradable costs were expressed in Kenyan Shillings, inflated to 2012 via Kenyan GDP deflator [33], and converted into USD at reference year exchange rates [40]. Ingredient costs considered in the marginal analysis include commodities, training and distribution. A sensitivity analysis was conducted for the intervention costs by varying costs −50%/+100% (**Table 3**).

**Table 3:** Costing and sensitivity analysis of malaria control interventions in Kenya.

					Sensitivity analysis	
Intervention	Unit	Distribution method	Economic cost per unit	Marginal economic cost per unit	Lower value	Upper value
**Long lasting insecticide-treated bednets (LLIN)**	Net delivered	Mass campaign through community organizations [55]	$8.52	$8.37	$4.26	$17.04
**Indoor residual spraying (IRS)**	Person protected	Annual mass campaign [55]	$0.73	$0.34	$0.34	$1.46
**School-based intermittent screen and treat (IST)**	Child screened	School-based distribution [56]	$6.32	$2.89	$3.16	$12.63

All costs are in 2012 USD.

### Analysis

#### Epidemiological outcomes

The simulated effectiveness of malaria control interventions and intervention combinations was evaluated by calculating the mean and inter-quartile range (IQR) of all model variants and seeds for each intervention combination for the difference in disease burden over a five year period from the start of intervention deployment compared to the mean of the simulations of the base case scenario with no interventions other than the existing case management system. Outcomes evaluated include decrease in parasite prevalence, number of uncomplicated episodes, hospitalizations and deaths averted in the general population. In addition to indicators for severity of illness, the overall population burden averted in terms of disability adjusted life years (DALYs) is calculated by combining mortality and morbidity measures as described by Murray and Lopez [25], [41]. Following standard methodology for cost effectiveness analysis presented by Drummond and colleagues [24], years of life lost to illness (YLLs) are calculated assuming age-specific life expectancies based on the life-table from Butajira, Ethiopia, with an average life expectancy of 46.6 years at birth [42].

#### Cost effectiveness calculation

Estimates of effectiveness of control interventions and intervention mixes are combined with the added costs of implementing these control measures. Treatment cost savings, or the reduction in cost to the health system due to the reduction in cases seen by the system, achieved by implementing the control strategy, are used to offset implementation costs and thus cost effectiveness ratios are calculated based on net rather than total intervention costs.

The cost savings to the case management system and households (CM) associated with implementing each intervention combination (IC) instead of a scenario without interventions (NO) are computed as DC_cmNO_−DC_cmIC_, where DC_cmNO_ are the direct costs (DC) of case management in the scenario without interventions and DC_cmIC_ are the direct costs of case management in the case of each intervention combination. These cost savings are subtracted from the direct cost of implementing each intervention combination (DC_int_) to give a net intervention combination cost (NC) computed as follows: NC = DC_int_−(DC_cmNO_−DC_cmIC_). Cost effectiveness is evaluated in two ways. The first is by calculating the average cost effectiveness ratio (ACER), as the net cost (NC) of the intervention divided by the net effects (NE) of the intervention. The second is by calculating the incremental cost effectiveness ratio (ICER), which follows the same methodology for calculating the ACER, except the net costs and net effects of each intervention combination are calculated against the currently implemented strategy.

Both marginal and average cost-effectiveness ratios over a five year reference period are reported to illustrate the likely short-term financial impact of the intervention, as well as the longer-term impact associated with the intervention including structural changes in health care delivery in response to lower disease burden achieved by the program. Cost effectiveness ratios are reported without discounting of future costs and benefits due to the short implementation time frame of the study and the recommendation from the revised GBD study [43]. Cost effectiveness ratios are calculated for a range of policy relevant outcomes including cost per case, hospitalization, death, and DALYs averted.

## Results

### Epidemiological outcomes

Compared to an intervention scenario with no malaria control outside of routine case management, and after five years of implementation, the intervention combination with LLIN use by 80% of the population, 90% of households covered by IRS with deployment starting in April, and IST of school children using AL with 80% coverage twice per term result in the largest simulated reduction in all-age parasite prevalence (99%, IQR 99.1–99.3%), average averted cases of uncomplicated malaria per person (7.46, IQR 7.44–7.48), hospitalizations averted (thousands)(3.96, IQR 3.95, 3.98), deaths averted (1,541, IQR 1,535, 1,551), and DALYs averted (thousands) (77.6, IQR 77.3–78.2) (**Table 4**).

**Table 4:** Simulated effect of intervention combinations.

	Proportion reduction in all-age parasite prevalence, year 5		Uncomplicated episodes averted per person		Hospitalizations averted (thousands)		Deaths averted		DALYs averted (thousands)	
	Mean	IQR	Mean	IQR	Mean	IQR	Mean	IQR	Mean	IQR
**Current strategy**										
LLIN 56%+IRS 70%	0.96	(0.95, 0.96)	7.04	(6.97, 7.10)	3.78	(3.74, 3.83)	1.42	(1.40, 1.44)	71.48	(70.77, 2.37)
**Increase coverage**										
LLIN 80%+IRS 90%	**0.99**	**(0.98, 0.99)**	**7.43**	**(7.40, 7.45)**	**3.96**	**(3.94, 3.97)**	**1.52**	**(1.51, 1.53)**	**76.27**	**(75.93, 6.75)**
**Add school-based IST**										
LLIN 56%+IRS 70%+IST 80% twice per term	**0.98**	**(0.97, 0.98)**	**7.16**	**(7.08, 7.22)**	**3.82**	**(3.79, 3.87)**	**1.46**	**(1.45, 1.48)**	**73.68**	**(73.07, 4.47)**
LLIN 80%+IRS 90%+IST 80% twice per term	**0.99**	**(0.99, 0.99)**	**7.46**	**(7.44, 7.48)**	**3.96**	**(3.96, 3.98)**	**1.54**	**(1.54, 1.55)**	**77.57**	**(77.26, 8.16)**
**Change timing of IRS**										
LLIN 56%+IRS 70% April start	**0.97**	**(0.96, 0.97)**	**7.09**	**(7.01, 7.15)**	**3.80**	**(3.76, 3.86)**	**1.43**	**(1.42, 1.45)**	**71.85**	**(71.27, 2.83)**
LLIN 56%+IRS 70% May start	0.95	(0.95, 0.96)	6.98	(6.90, 7.05)	3.75	(3.71, 3.82)	1.40	(1.39, 1.42)	70.48	(69.81, 1.50)
LLIN 56%+IRS 70% June start	0.96	(0.96, 0.97)	**7.08**	**(7.01, 7.13)**	**3.80**	**(3.77, 3.84)**	**1.43**	**(1.41, 1.44)**	**71.79**	**(71.09, 2.80)**
**Change timing and increase coverage of IRS**										
LLIN 56%+IRS 90% April start	0.76	(0.73, 0.78)	6.12	(5.85, 6.25)	3.26	(3.06, 3.45)	1.21	(1.14, 1.27)	61.31	(58.17, 4.18)
LLIN 56%+IRS 90% May start	**0.97**	**(0.96, 0.97)**	**7.07**	**(7.01, 7.13)**	**3.79**	**(3.75, 3.84)**	**1.43**	**(1.41, 1.45)**	**71.79**	**(71.19, 2.88)**
LLIN 56%+IRS 90% June start	**0.97**	**(0.97, 0.98)**	**7.16**	**(7.09, 7.22)**	**3.84**	**(3.81, 3.88)**	**1.45**	**(1.43, 1.46)**	**72.78**	**(72.20, 3.63)**
**IRS alone, change coverage**										
IRS 70%	0.53	(0.50, 0.55)	2.89	(2.06, 3.33)	1.66	(1.35, 1.97)	0.60	(0.49, 0.74)	30.33	(25.56, 7.05)
IRS 90%	0.66	(0.63, 0.67)	3.63	(2.95, 3.99)	2.10	(1.86, 2.37)	0.74	(0.66, 0.85)	37.62	(33.56, 2.63)
**LLINs alone**										
LLIN 56%	0.95	(0.95, 0.96)	7.00	(6.93, 7.05)	3.76	(3.72, 3.82)	1.41	(1.39, 1.43)	70.86	(70.11, 1.95)
LLIN 80%	0.94	(0.93, 0.94)	**7.15**	**(7.08, 7.22)**	**3.80**	**(3.76, 3.86)**	**1.44**	**(1.42, 1.46)**	**72.65**	**(71.76, 3.68)**
**School-based IST alone**										
IST 40% once per term	0.09	(0.05, 0.16)	0.28	(−0.83, 1.05)	0.16	(−0.17, 0.63)	0.07	(−0.04, 0.26)	3.40	(−2.12, 12.75)
IST 40% twice per term	0.14	(0.11, 0.20)	0.46	(−0.66, 1.20)	0.25	(−0.08, 0.68)	0.10	(−0.001, 0.32)	5.21	(−0.33, 14.80)
IST 80% once per term	0.16	(0.13, 0.21)	0.53	(−0.59, 1.27)	0.29	(−0.05, 0.74)	0.12	(−0.01, 0.35)	5.93	(−0.84, 16.08)
IST 80% twice per term	0.22	(0.19, 0.27)	0.78	(−0.27, 1.46)	0.42	(0.08, 0.82)	0.16	(0.05, 0.36)	8.14	(2.34, 18.06)

Compared to a scenario with no interventions outside the existing case management system, the mean and inter-quartile range of the impact of different intervention combinations (**Table 1**) on epidemiological outcomes in a population of 100,000 individuals over a time period of five years*. **Bold** figures indicate mean results improved from the current strategy.

*Unless otherwise indicated.

**Figure 1:**
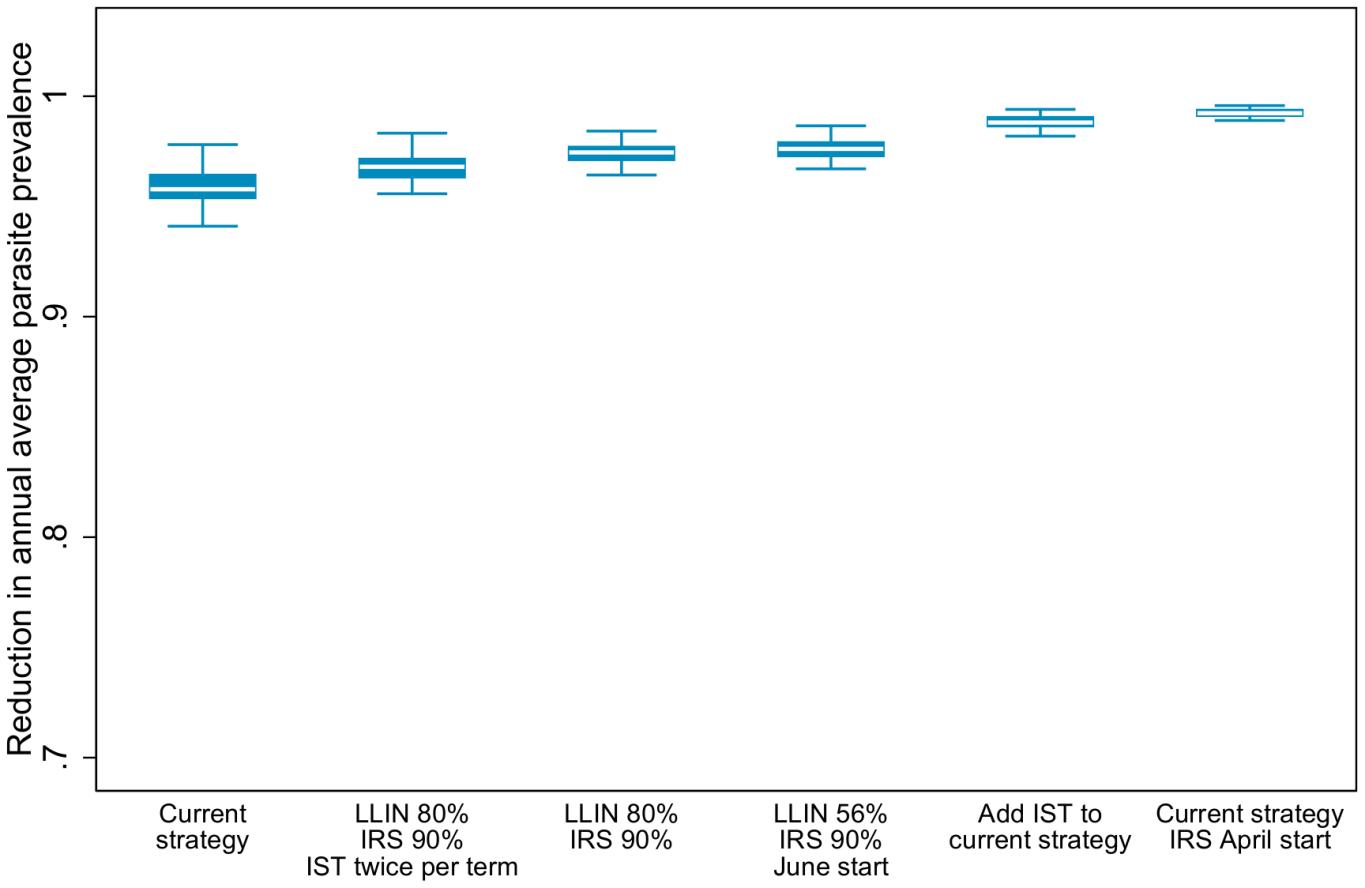
Simulated reduction in all-age annual average parasite prevalence by intervention combination compared to a scenario with no intervention. White lines represent the simulated median value, blue boxes represent the inter-quartile range, and capped bars represent the upper and lower adjacent values for simulated results for each intervention combination using an ensemble of 14 model variants and five random seeds. Choice of intervention combinations is based on the criteria of simulated reduction in parasite prevalence greater than the strategy currently implemented in the study area.

Simulation results indicate that increased coverage of vector control has a larger impact than adding an IST intervention to the current control strategy. However, adding the highest IST coverage and frequency to the current strategy could reduce parasite prevalence by an additional nine percentage points (**Figure 1**). Despite high coverage levels of all interventions, the scenario with the largest simulated epidemiological impact only resulted in one fewer uncomplicated case per person over the course of five years compared to the level observed in the study area with the current strategy (**Table 4**). Changing the timing of IRS deployment did not result in a reduction in simulated parasite prevalence either at observed coverage levels or when coverage was increased to 90% (**Table 4**).

**Figure 2:**
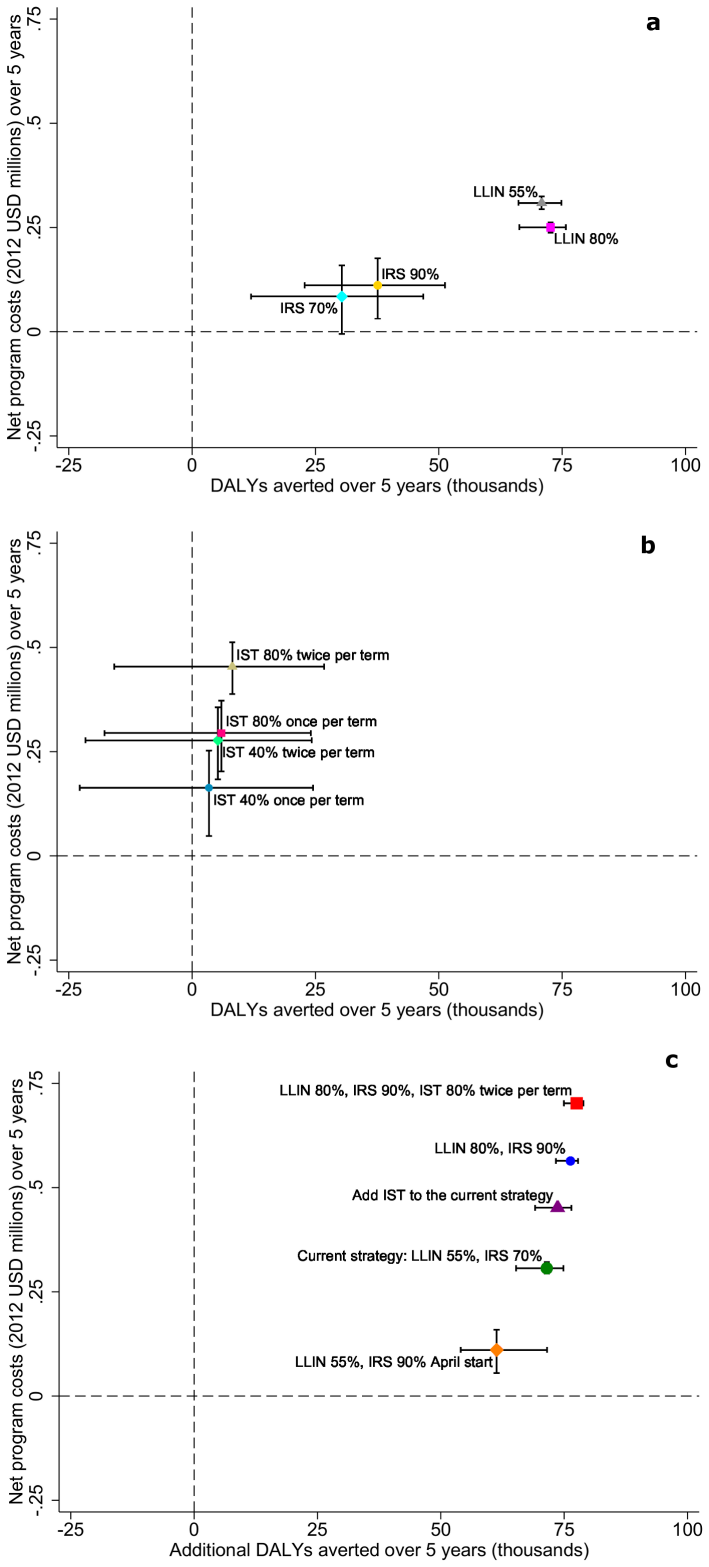
Relationship between cost and simulated health impact. Simulated cumulative DALYs averted after five years compared to the no intervention scenario by net program costs for different implementation strategies of **a**) vector control interventions, **b**) intermittent screen and treat in school children, and **c**) combinations of interventions. Symbols represent the mean simulation results across 14 model variants and five random seeds. Horizontal capped bars represent range of simulated DALYs averted. Vertical capped bars represent range of simulated net program costs. Negative DALYs averted indicate simulated interventions that have a worse health outcome than the no intervention scenario. Negative net program costs indicate simulated interventions where the savings to the health system are greater than the delivery costs.

Despite moderate levels of self-reported LLIN use, simulations indicate LLINs, and not IRS, account for the majority of impact on parasite prevalence. Removing LLINs and continuing only with a higher level of IRS coverage resulted in a similar number of averted uncomplicated cases compared to the IST interventions (**Table 4**). With higher LLIN use, simulations indicate IRS adds only a limited additional benefit above that provided by the nets (**Figure 1** **,** **Figure 2c**).

Depending on coverage level and frequency, without vector control interventions, simulations suggest IST could reduce annual average parasite prevalence in the population by 9–22% (**Table 4**). In the absence of vector control interventions, when starting with the IST 40% coverage once per term scenario, and compared to a scenario with no interventions, keeping the same coverage and increasing doses to twice per term showed a similar reduction in parasite prevalence as keeping the same frequency and increasing IST coverage to 80% (**Figure 2**).

### Costing

**Figure 3:**
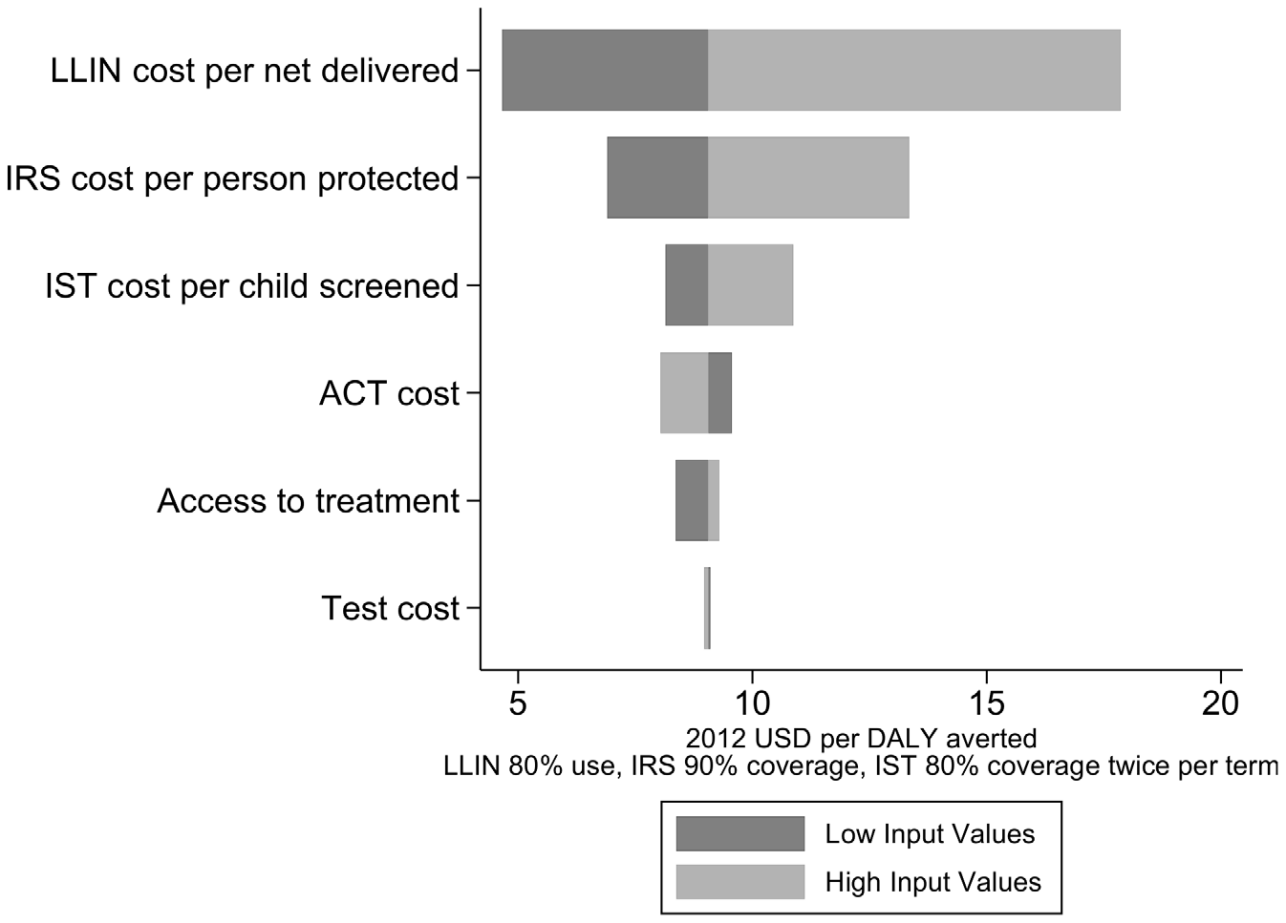
Sensitivity analysis. Tornado diagram of the change in the ACER of an intervention with 80% LLIN use, 90% IRS coverage, and 80% IST coverage twice per term in relation to variation in component costs.

Total delivery costs and net health system costs for implementing each intervention combination can be found in **Table S1**. Program costs always exceeded savings in case management. The top contributor to uncertainty in the highest coverage intervention combination scenario was the cost per LLIN distributed, followed by cost per child screened, ACT cost, cost per person protected by IRS, and access to treatment (**Figure 3**). Because of a low proportion of fevers tested for malaria with an RDT (12%), test cost did not contribute greatly to overall uncertainty.

### Intervention combination cost effectiveness

**Figure 4:**
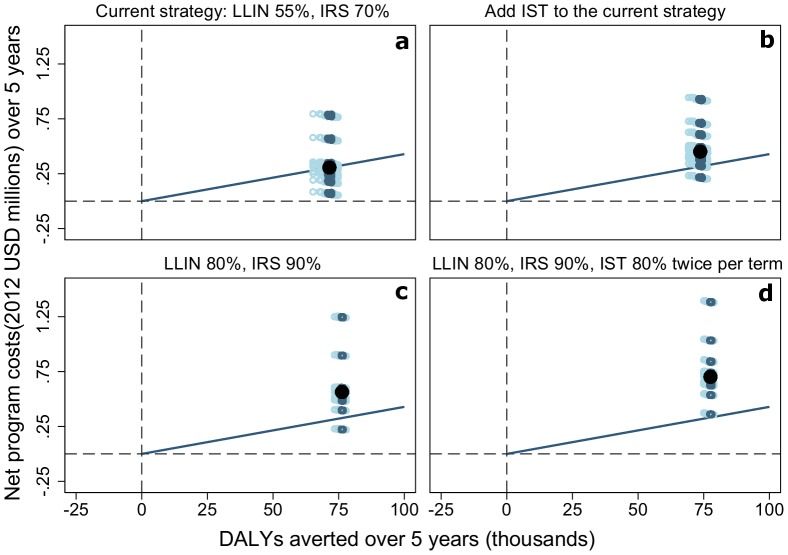
Cost effectiveness planes. Simulated cumulative DALYs averted in a population of 100,000 individuals after five years compared to the no intervention scenario by net program costs for the intervention combinations with a better simulated health outcome than the currently implemented malaria control strategy, ranked in descending order of ACER. Black dots represent the mean simulation results across 14 model variants and five seeds. Circles represent the of simulated DALYs averted by net program costs with different assumptions of input costs of the case management system and malaria control interventions in the study area represented in Table 2 and Table 3. Dark blue circles are within the inter-quartile range of simulated DALYs averted and light blue circles are outside the range. Negative DALYs averted indicate simulated interventions that have a worse health outcome than the no intervention scenario. Negative net program costs indicate simulated interventions where the savings to the health system are greater than the delivery costs. Diagonal lines correspond to the ratios of mean (4.29 USD per DALY averted) ACER of the currently implemented intervention combination in the study area (LLIN use 56%, IRS coverage 70%).

Five intervention combinations simulated more averted DALYs than the currently-implemented intervention combination (**Table 4** **,** **Figure 4**). All of these intervention combinations involve increasing coverage of LLINs, of IRS, or both, with the exception of one which adds IST to the current strategy (**Table 4** **,** **Figure 4**). However, none of these options were simulated to be more cost effective than the current strategy (**Table 5**). All interventions can be considered very cost effective health interventions. The currently implemented intervention combination has a simulated ACER of 4.29 USD per DALY averted, but even the intervention combination with the highest cost per additional DALY averted, IST at 80% coverage twice per term, has a simulated ACER of only 55.70 USD (**Table 5**).

**Table 5:** Cost effectiveness of different intervention combinations for a population of 100,000 over five years of implementation (2012 US$).

	Average ACER		Marginal ACER		Average ICER	Marginal ICER
	*Mean*	*IQR*	*Mean*	*IQR*	*Mean*	*Mean*
**Current strategy**						
Current strategy: LLIN 55%, IRS 70%	4.29	(4.22, 4.33)	6.30	(6.28, 6.31)		
**Change timing and increase coverage of IRS**						
LLIN 55%, IRS 90% May start	5.27	(5.21, 5.31)	6.75	(6.74, 6.75)	235.46	111.58
LLIN 55%, IRS 90% June start	5.11	(5.06, 5.13)	6.62	(6.61, 6.62)	50.24	24.27
**Add IST**						
Add IST to the current strategy	6.13	(6.09, 6.14)	7.02	(7.01, 7.03)	66.03	30.55
**Increase coverage**						
LLIN 80%, IRS 90%	7.39	(7.38, 7.40)	8.92	(8.92, 8.92)	53.75	48.06
**Add IST, increase coverage**						
LLIN 80%, IRS 90%, IST 80% twice per term	9.06	(9.04, 9.05)	9.59	(9.58, 9.60)	65.05	48.27

The mean and inter-quartile range of the average cost effectiveness ratios (ACER) compared to a scenario with no interventions outside the existing case management system, and incremental cost effectiveness ratios (ICER) compared to the currently implemented strategy for different intervention combinations with more simulated DALYs averted than the currently implemented strategy. ACERs and ICERs are calculated using costs reported in **Table S1** and effectiveness reported in **Table 1**. Interventions are displayed in ascending order of simulated DALYs averted (**Table 1**). IQR represents mean costs values applied to the inter-quartile range of simulated health effects.

## Discussion

Cost effectiveness analyses based on health outcomes simulated by transmission models can compare many more intervention effects than can static models or field trials. In these simulations, interventions simulate a decrease in vector population and a corresponding decrease in transmission that allows for mass community effects of interventions. In particular, such models can explore the effects of intervention scenarios by transmission level and coverage level whereas in single field studies all the effects of different interventions cannot be captured.

Increased coverage and use of vector control interventions has a larger simulated impact on all malaria indicators than adding IST to the currently implemented control strategy. There could be additional impact of IST programs not captured in this analysis, including improved school performance and decreased anemia [44]. While results from a cluster-randomized trial of once per term IST in school children in the south coast of Kenya at similar coverage levels did not show an impact on parasitaemia [45], effectiveness of the program will depend on baseline parasitaemia and results may be different in Rachuonyo South District. Results suggests that, at least at transmission levels comparable to those in the study area, it would not be warranted to take focus away from vector control in favor of a school-based IST program even at a deployment frequency of twice per term, assuming such a level exists where this would be advisable. The simulated screen and treat campaign in this study was limited to school children, and incorporating a focal- or mass screen and treat program in the community may have very different results. However, should Rachuonyo South District decide to implement an IST program, simulations indicate adding this intervention to the existing malaria control program could still be a cost-effective intervention with a mean simulated ICER of only 66 USD above the currently implemented strategy (**Table 5**).

Despite moderate observed use in the population, simulations show LLINs and not IRS account for the majority of impact on disease burden. Changing the timing of IRS implementation did not have a large impact on parasite prevalence. This could be due to the simulation experiment design, which models implementation of IRS programs rolled out over a 60 day period culminating in the target proportion of individuals protected. Because the start date of implementation was varied by 30 days at a time, implementation could overlap enough to prevent observing a substantial difference between scenarios. Rather than changing the timing or coverage of IRS, the study area may benefit from adding new vector control interventions, particularly those targeting exophagic and exophilic vectors.

The simulation results for the effect of the currently-implemented strategy on parasite prevalence in the study area have been previously validated and found to be in the range of the effects observed in the field [4]. The large simulated reduction in parasite prevalence compared to a case-management only strategy in many of the simulated intervention combinations described in **Figure 2** can be attributed to high coverage of interventions over an extended period of time, conditions which may or not be operationally sustainable.

### Limitations

While interventions were chosen to correspond to those in the hotspot-targeted intervention study, simulated implementation was assumed for the whole population rather than target hot spots because OpenMalaria does not incorporate an explicit spatial element. Therefore results cannot be matched against intervention trial results for validation purposes. However, findings from this experiment can help put the trial results in the broader context of what could be expected from community-wide implementation of combinations of interventions.

While simulations of the scenarios describing the effects of the intervention combinations in reducing malaria burden account for uncertainty by employing an ensemble of 14 model variants and multiple random seeds, uncertainty in the costing model is limited to a one-way sensitivity analysis. A probabilistic sensitivity analysis exposing the model to changes in assumptions of inputs to the case management and intervention unit costs is being conducted for publication elsewhere, and will assist in clarifying the uncertainty inherent in these predictions.

Despite vector behavior in the study area favoring outdoor biting, IRS had a lower health impact than expected when simulated as a stand-alone intervention when compared to LLINs. The IRS model parameterization has deterrency and killing effects of half that of LLINs, due to simulated action only on post-prandial indoor resting mosquitoes, in contrast to the both pre- and post- prandial killing effect of LLINs. A model update will allow the effect of IRS to be simulated on both states of the mosquito feeding cycle, and the parameters for effectiveness of IRS should be updated based on experimental hut data. It is also worth noting the lower cost per sachet of insecticide assumed in the costing model compared to the average unit costs reported in the recently released UNITAID report on malaria vector control commodities [46], due to the economies of scale achieved through a multi-country procurement by the IRS implementing partner [47].

### Implications of results for health systems

Results of this experiment have the potential to assist malaria control program managers in the study area in deciding on adding new or changing the implementation of current interventions. All the simulated intervention combinations can be considered cost effective in the context of levels of health expenditure in Kenya. Malaria is the number six contributor to the burden of disease in Kenya, both overall and in children under five [48]. The low cost per DALY averted by the malaria control interventions with a higher simulated number of DALYs averted than the current strategy represents a small portion of the total health expenditure per capita of 42 USD [13] and could be a cost effective option for reaching the country's development strategies. In comparison with estimates from a recent systematic review on costs and cost effectiveness of malaria control interventions [39], these results are on the low end of the range of previous estimates. Similarly, compared to WHO-CHOICE estimates for the AFR-E region, while the simulated DALYs averted per year for the currently-implemented strategy are comparable to WHO estimates for 50% coverage of vector control interventions (14,296 simulated, 14,711 observed), the simulated cost per DALY averted are substantially lower than the regional averages when converted to 2012 USD (4.29 2012 USD simulated, 50 2005 International Dollars (I$) observed) [49]. This puts malaria prevention interventions in the study area in the range of regional estimates for tuberculosis (6–15 2005 I$ per DALY averted) [50] and HIV prevention communication (3–4 2005 I$ per DALY averted) [51].

Findings from this study indicate there are several combinations of interventions that could result in a greater health impact per dollar spent than the currently implemented strategy in the study area. However, increasing LLIN use and IRS coverage and initiating a school-based IST program will require investment in several elements not included in this analysis. Firstly, the unit costs of scaling up or introducing some programs will vary by implementation strategy more than others. For example, the majority of the economic cost of the LLIN program implemented by training existing community organizations on distribution is represented by the marginal cost of procuring nets (**Table 3**). Therefore a change in strategy may not result in a large change in cost per net delivered due to increased or decreased non-commodity costs. The reverse is true for a school-based IST program where marginal costs are under half the cost per child screened (**Table 3**), and could therefore be far more sensitive to changes in program design.

Secondly, additional costs will be incurred by determining the appropriate strategy for achieving programmatic goals. Several scenarios in this experiment assume LLIN use of 80%, which is an ambitious target that will depend not only on universal coverage but a large behavior change communications component. Understanding of the behavioral determinants for why nets existing in households currently remain unused will be critical to achieving this goal. In addition to increased personnel and commodities, increasing coverage of IRS will require continued monitoring of insecticide resistance in the vector population, as well as understanding why households remain unsprayed, whether it is due to rejection by household members or the inability to logistically access hard to reach households. Implementing a school-based IST program as intensive as twice per school term over an extended period of time could result in a change in adherence rates as well as an increased risk of selecting for drug resistance, elements which may impact the effectiveness of the intervention if community acceptability is not assessed.

Thirdly, the study does not allow for any economies or diseconomies of scale for the costs of commodities and program delivery, assuming costs will grow linearly with scale up. In practice this will likely not be the case; increasing intervention coverage from 70% to 80% may be more expensive than scaling up from 50% to 60%.

Assessing the epidemiologic impact and cost effectiveness of different intervention combinations is a necessary element in considering a change of malaria control policy, but it is by no means the only criteria with which to base a recommendation for policy change. Changes in implementation, whether this includes new strategies to increase coverage and use of existing interventions or the addition of a new intervention, will have implications on acceptability by the individuals and communities receiving the interventions, the personnel involved in service delivery, the natural environment into which additional insecticides could be introduced, and the systems of surveillance and monitoring for indicators of malaria and other febrile illnesses, to name a few. Conducting a health impact assessment, drawing on existing frameworks [52], [53], may strengthen the success of any change in strategy.

## Supporting Information

Table S1 **Total costs and cost savings of different intervention combinations for a population of 100,000 over five years of implementation.**
(DOCX)

Text S1 **Costing the Kenya malaria case management system and interventions.** Contains Tables A–C.
(DOCX)
