# Supplementary material for: Modeling the Cost Effectiveness of Malaria Control Interventions in the Highlands of Western Kenya
Source: PLoS One. 2014 Oct 7;9(10):e107700. doi: 10.1371/journal.pone.0107700 (PMC4188563; doi:10.1371/journal.pone.0107700)
Supplement: Table S1 — Total costs and cost savings of different intervention combinations for a population of 100,000 over five years of implementation. (DOCX) [file pone.0107700.s001.docx]

**Table S1: Total costs and cost savings of different intervention combinations for a population of 100,000 over five years of implementation (2012 US$)**

|  | Total intervention costs (thousands) | | | | Net program costs (thousands) | | | |
| --- | --- | --- | --- | --- | --- | --- | --- | --- |
|  | Average | | Marginal | | Average | | Marginal | |
|  | Mean | IQR | Mean | IQR | Mean | IQR | Mean | IQR |
| **Current strategy** |  |  |  |  |  |  |  |  |
| LLIN 55.63% + IRS 70% | 732.54 | (731.60, 733.21) | 587.23 | (586.29, 587.94) | 306.42 | (309.52, 301.68) | 450.17 | (450.93, 448.93) |
| **Increase coverage** |  |  |  |  |  |  |  |  |
| LLIN 80% + IRS 90% | 1014.21 | (1013.47, 1014.87) | 826.04 | (825.32, 826.64) | 564.00 | (564.23, 562.91) | 680.49 | (680.16, 680.46) |
| **Add IST** |  |  |  |  |  |  |  |  |
| LLIN 55.63% + IRS 70% + IST 80% twice per term | 884.93 | (882.85, 886.09) | 657.13 | (656.05, 657.94) | 451.59 | (452.73, 448.63) | 517.34 | (517.45, 516.71) |
| LLIN 80% + IRS 90% + IST 80% twice per term | 1154.39 | (1153.03, 1155.64) | 890.31 | (889.41, 891.25) | 702.44 | (701.58, 702.36) | 744.06 | (743.44, 744.50) |
| **Change timing of IRS** |  |  |  |  |  |  |  |  |
| LLIN 55.63% + IRS 70% April start | 806.81 | (805.63, 807.85) | 622.29 | (621.21, 623.31) | 377.39 | (379.12, 373.81) | 484.06 | (484.26, 483.53) |
| LLIN 55.63% + IRS 70% May start | 732.59 | (731.60, 733.56) | 587.27 | (586.33, 588.22) | 310.43 | (314.45, 304.52) | 451.58 | (452.43, 450.21) |
| LLIN 55.63% + IRS 70% June start | 732.81 | (731.43, 734.05) | 587.49 | (586.10, 588.66) | 303.94 | (306.63, 300.55) | 449.47 | (449.82, 448.99) |
| **Change timing and increase coverage of IRS** |  |  |  |  |  |  |  |  |
| LLIN 55.63% + IRS 90% April start | 474.27 | (473.20, 475.18) | 465.91 | (464.85, 466.81) | 110.76 | (128.79, 97.26) | 350.38 | (357.94, 346.37) |
| LLIN 55.63% + IRS 90% May start | 806.66 | (805.73, 807.74) | 622.14 | (621.24, 623.22) | 378.53 | (381.11, 374.27) | 484.34 | (484.72, 483.57) |
| LLIN 55.63% + IRS 90% June start | 806.21 | (805.07, 806.97) | 621.71 | (620.58, 622.47) | 371.82 | (373.65, 368.32) | 481.75 | (482.09, 480.97) |
| **IRS alone, change coverage** |  |  |  |  |  |  |  |  |
| IRS 70% | 258.50 | (258.42, 258.59) | 121.53 | (121.50, 121.58) | 84.36 | (124.58, 55.88) | 68.00 | (83.86, 57.26) |
| IRS 90% | 332.45 | (332.38, 332.52) | 156.30 | (156.27, 156.34) | 111.29 | (148.43, 82.71) | 87.83 | (102.31, 78.02) |
| **LLINs alone** |  |  |  |  |  |  |  |  |
| LLIN 55.63% | 732.53 | (731.70, 733.43) | 587.20 | (586.47, 588.12) | 308.88 | (313.14, 304.45) | 450.97 | (451.85, 449.97) |
| LLIN 80% | 682.05 | (681.41, 682.80) | 670.03 | (669.40, 670.76) | 250.58 | (254.95, 246.24) | 530.95 | (532.55, 529.93) |
| **IST alone** |  |  |  |  |  |  |  |  |
| IST 40% once per term | 179.14 | (171.79, 184.35) | 81.98 | (78.62, 84.36) | 163.31 | (207.20, 123.63) | 77.32 | (94.88, 61.24) |
| IST 40% twice per term | 302.59 | (290.01, 311.97) | 138.47 | (132.71, 142.76) | 277.25 | (311.14, 242.31) | 130.90 | (146.33, 116.94) |
| IST 80% once per term | 323.87 | (310.19, 333.80) | 148.21 | (141.95, 152.75) | 294.53 | (325.73, 4.3401) | 139.46 | (154.61, 26.01) |
| IST 80% twice per term | 497.76 | (477.02, 516.82) | 227.78 | (218.30, 236.51) | 454.06 | (477.80, 433.41) | 214.38 | (226.50, 206.64) |
