# Supplementary material for: Modeling the Cost Effectiveness of Malaria Control Interventions in the Highlands of Western Kenya
Source: PLoS One. 2014 Oct 7;9(10):e107700. doi: 10.1371/journal.pone.0107700 (PMC4188563; doi:10.1371/journal.pone.0107700)
Supplement: Text S1 — Costing the Kenya malaria case management system and interventions. Contains Tables A–C. (DOCX) [file pone.0107700.s002.docx]

**Text S1: Costing the Kenya malaria case management system, interventions, and cost effectiveness**

**Table A:** Costing model parameterization of access to and cost of the Kenya case management system

| **Parameter** | **Value** | | **Cost (2012 USD)** | |
| --- | --- | --- | --- | --- |
| Proportion of the most recent episode of fevers within 2 week recall seeking medical care | 0.618 [[1](#_ENREF_1)] |  | |  |
| Proportion of fevers treated at a formal health facility | 0.486 [[1](#_ENREF_1)] |  | |  |
| *Health center without beds* | 0.556 [[1](#_ENREF_1)] | 1.59 [[2](#_ENREF_2)] | |  |
| *Health center with beds* | 0.209 [[1](#_ENREF_1)] | 1.97 [[2](#_ENREF_2)] | |  |
| *Hospital outpatient* | 0.235 [[1](#_ENREF_1)] | 2.29 [[2](#_ENREF_2)] | |  |
| *Hospital inpatient* |  | 6.96 [[2](#_ENREF_2)] | |  |
| Coartem (ACT) | 0.439 |  | |  |
| *(6 x 2 tablets co-pack) 20mg + 120mg tab-cap* | **Table A2** | 0.0898*[[3](#_ENREF_3)] | |  |
| Proportion of ACT treatments with full adherence | 0.771 |  | |  |
| Quinine (QN) |  |  | |  |
| *Intravenous (IV), Day 1* | **Table A2** | 0.1324* [[3](#_ENREF_3)] | |  |
| *Intravenous (IV), Day 2* | **Table A2** |  | |  |
| *Oral, Day 3* | **Table A2** | 0.0524* [[3](#_ENREF_3)] | |  |
| Sulfadoxine-Pyrimethamine (SP) | 0.214 | 0.0299* [[3](#_ENREF_3)] | |  |
| Proportion of children under 5 with fever tested for malaria in any setting |  |  | |  |
| *Paracheck® rapid diagnostic test (RDT)* | 0.12 [[4](#_ENREF_4)] | 0.62 [[5](#_ENREF_5)] | |  |
| Cost of travel to seek treatment |  | 0.40** | |  |
| Cost of consumables while seeking treatment |  | 0.20 [[6-8](#_ENREF_6)] | |  |
| *Median supplier price **Multi-country literature review | | | |  |

**Table B:** Drug dosing per full course per age group for treatment of uncomplicated and severe illness as per 2010 WHO malaria treatment guidelines [[9](#_ENREF_9)]

| **Years of age** | **ACT dose** | **Quinine Dose** | | | |
| --- | --- | --- | --- | --- | --- |
|  | *20mg + 120mg tab-cap* | | *Day 1 (IV, per 300 ampule)* | *Day 2 (IV, per 300 ampule)* | *Day 3 (oral, per 300mg tablet)* |
|  |  | | 20 mg/kg over 4 hours | 10 mg/kg every 8 hours | 10 mg/kg every 8 hours |
| **<1** | 6 | | 180 | 270 | 270 |
| **1 - 3** | 6 | | 240 | 360 | 360 |
| **3 - 5** | 12 | | 360 | 540 | 540 |
| **5 - 6** | 12 | | 420 | 630 | 630 |
| **6 - 10** | 12 | | 600 | 900 | 900 |
| **10 – 12** | 18 | | 840 | 1260 | 1260 |
| **12 - 16** | 24 | | 1080 | 1620 | 1620 |
| **> 16** | 24 | | 1200 | 1800 | 1800 |

**Table C:** Source for methodology followed in the costing exercise

| **Methodology** | **Source** |
| --- | --- |
| Costing of case management system | Tediosi et al. [[6](#_ENREF_6)] |
| Costing of malaria control interventions | Kolaczinski et al. [[10](#_ENREF_10)], White et al. [[11](#_ENREF_11)] |
| Calculating the burden of disease (DALYs) | Murray and Lopez [[12](#_ENREF_12),[13](#_ENREF_13)] |
| Cost-effectiveness analysis | Drummond et al. [[14](#_ENREF_14)] |

**References**

1. Kenya National Bureau of Statistics, ICF Macro (2010) Kenya Demographic and Health Survey 2008-09. Calverton, Maryland.

2. WHO-CHOICE (2008) Unit Costs Estimates for Service Delivery - Estimation File. Geneva.

3. Management Sciences for Health (2011) International Drug Price Indicator Guide. Cambriage, MA: MSH.

4. Department of Malaria Control, Kenya Bureau of Statistics, ICF Macro (2011) 2010 Kenya Malaria Indicator Survey. Nairobi, Kenya.

5. Global Fund to Fight AIDS, TB and Malaria (2013) Price and Quality Reporting Tool.

6. Tediosi F, Maire N, Smith T, Hutton G, Utzinger J, et al. (2006) An approach to model the costs and effects of case management of Plasmodium falciparum malaria in sub-saharan Africa. Am J Trop Med Hyg 75: 90-103.

7. Batwala V, Magnussen P, Hansen KS, Nuwaha F (2011) Cost-effectiveness of malaria microscopy and rapid diagnostic tests versus presumptive diagnosis: implications for malaria control in Uganda. Malar J 10: 372.

8. Malaria Vaccine Initiative (2012) Estimating the economic burden of malaria in Nigeria. Malaria Vaccine Initiative.

9. WHO (2010) Guidelines for the Treatment of Malaria, Second Edition. Geneva.

10. Kolaczinski J, Hanson K (2006) Costing the distribution of insecticide-treated nets: a review of cost and cost-effectiveness studies to provide guidance on standardization of costing methodology. Malar J 5: 37.

11. White MT, Conteh L, Cibulskis R, Ghani AC (2011) Costs and cost-effectiveness of malaria control interventions--a systematic review. Malar J 10: 337.

12. Murray CJ, Lopez AD (1996) The global burden of disease: a comprehensive assessment of mortality and disability from diseases, injuries, and risk factors in 1990 to 2020. Cambridge, MA, USA: Harvard University Press.

13. Murray CJ, Vos T, Lozano R, Naghavi M, Flaxman AD, et al. (2012) Disability-adjusted life years (DALYs) for 291 diseases and injuries in 21 regions, 1990-2010: a systematic analysis for the Global Burden of Disease Study 2010. Lancet 380: 2197-2223.

14. Drummond MF, Sculpher M, Torrance G, O'Brien B, Stoddart G (2005) Methods for the Economic Evaluation of Health Care Programmes 3 ed. Oxford, United Kingdom: Oxford University Press.
